# Identification of inactive conformation‐selective interleukin‐2‐inducible T‐cell kinase (ITK) inhibitors based on second‐harmonic generation

**DOI:** 10.1002/2211-5463.12489

**Published:** 2018-07-30

**Authors:** Yoshiji Hantani, Kiyosei Iio, Rie Hantani, Kayo Umetani, Toshihiro Sato, Tracy Young, Katelyn Connell, Sam Kintz, Joshua Salafsky

**Affiliations:** ^1^ Biological/Pharmacological Research Laboratories Central Pharmaceutical Research Institute Japan Tobacco Inc. Takatsuki Osaka Japan; ^2^ Chemistry Research Laboratories Central Pharmaceutical Research Institute Japan Tobacco Inc. Takatsuki Osaka Japan; ^3^ Biodesy, Inc. South San Francisco CA USA

**Keywords:** biosensor, drug discovery, inactive kinase, interleukin‐2‐inducible T‐cell kinase, second‐harmonic generation, surface plasmon resonance

## Abstract

Many clinically approved protein kinase inhibitors stabilize an inactive conformation of their kinase target. Such inhibitors are generally highly selective compared to active conformation inhibitors, and consequently, general methods to identify inhibitors that stabilize an inactive conformation are much sought after. Here, we have applied a high‐throughput, second‐harmonic generation (SHG)‐based conformational approach to identify small molecule stabilizers of the inactive conformation of interleukin‐2‐inducible T‐cell kinase (ITK). A single‐site cysteine mutant of the ITK kinase domain was created, labeled with an SHG‐active dye, and tethered to a supported lipid bilayer membrane. Fourteen tool compounds, including stabilizers of the inactive and active conformations as well as nonbinders, were first examined for their effect on the conformation of the labeled ITK protein in the SHG assay. As a result, inactive conformation inhibitors were clearly distinguished from active conformation inhibitors by the intensity of SHG signal. Utilizing the SHG assay developed with the tool compounds described above, we identified the mechanism of action of 22 highly selective, inactive conformation inhibitors within a group of 105 small molecule inhibitors previously identified in a high‐throughput biochemical screen. We describe here the first use of SHG for identifying and classifying inhibitors that stabilize an inactive vs. an active conformation of a protein kinase, without the need to determine costructures by X‐ray crystallography. Our results suggest broad applicability to other proteins, particularly with single‐site labels reporting on specific protein movements associated with selectivity.

**Abbreviations**
cps: counts per second
HTS: high‐throughput screening
IL: interleukin
ITK: interleukin‐2‐inducible T‐cell kinase
KD: kinase domain
MOA: mechanism of action
NHS: *N*‐hydroxysuccinimide
SHG: second‐harmonic generation
SPR: surface plasmon resonance
WT: wild‐type

Protein kinases are the largest enzyme family encoded by the human genome and are comprised of over 500 members 1. Pharmacological agents directed to them have high potential to treat a number of diseases, spurring numerous lines of research 2. The overall structure of the catalytic domain of protein kinases is highly conserved 3. The sequence and the structural topologies of ATP‐binding sites of kinases in the active conformation are highly similar, with key residues optimally aligned for catalysis 4. Accordingly, the conserved ATP‐binding pocket creates significant difficulties for designing selective kinase inhibitors 5. In physiological conditions, most kinases exist in equilibrium between active and inactive conformational states 4. Inhibitors that stabilize an inactive conformation generally tend to show higher selectivity 6. High‐throughput screens (HTS) of protein kinases are commonly employed to identify kinase inhibitor candidates. These screens provide hits that inhibit the kinase activity but do not report on the resulting conformational state of the target. Thus, it is important for selectivity considerations to further identify those compounds from the HTS hits that stabilize inactive conformations before moving to crystallography. Second‐harmonic generation (SHG) has emerged as a technique for real‐time detection and measurement of protein conformational changes in solution and is broadly applicable across many target classes 7, 8, 9, 10, 11. The method is based on tethering proteins labeled with an SHG‐active dye to a surface such as a supported lipid bilayer membrane. SHG is highly sensitive to the orientation of the SHG‐active molecules, permitting the study of conformational changes in the target protein (Fig. 1).

**Figure 1:**
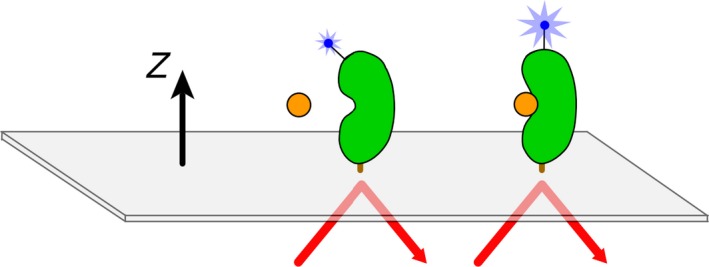
Schematic for protein conformational change detection by SHG. Incident laser light strikes the surface and through total internal reflection creates an evanescent wave. Labeled protein is bound to the surface, and the measured SHG signal intensity depends on the average, net orientation of the dye label relative to the surface normal (*z*‐axis). A conformational change that alters the orientational distribution of the label in space or time results in a change in SHG signal intensity. A decrease in SHG signal intensity is caused by the dye moving more perpendicular to the *z*‐axis, while an increase in SHG signal intensity is caused by the dye moving more parallel to the *z*‐axis. In this way, different conformational states of a protein produce different SHG signals.

In this report, we have developed an SHG assay for selection of inactive conformation inhibitors to Tec family tyrosine kinase, interleukin (IL)‐2‐inducible T‐cell kinase (ITK). ITK is expressed in T cells, mast cells, and NK cells, and it plays an important role in the production of cytokines such as IL‐2, IL‐4, IL‐5, and IL‐13 12, 13. Due to this role of ITK in T cells, inhibition of the kinase could offer the potential for treating T‐cell‐mediated inflammatory diseases 14, 15, 16.

## Materials and methods

### Materials

Biacore T200 and 4000 optical biosensors and NTA sensor chips were obtained from GE Healthcare (Little Chalfont, Buckinghamshire, UK).

ADP‐Glo™ Kinase Assay kit which contains ADP‐Glo™ Reagent, kinase detection reagent, ATP, and ADP was purchased from Promega (Madison, WI, USA).

Biodesy Delta 384‐well plates and the SHG‐active dye, SHG2‐maleimide (thiol reactive), were made available by Biodesy, Inc.

### Tool compounds

**Figure 2:**
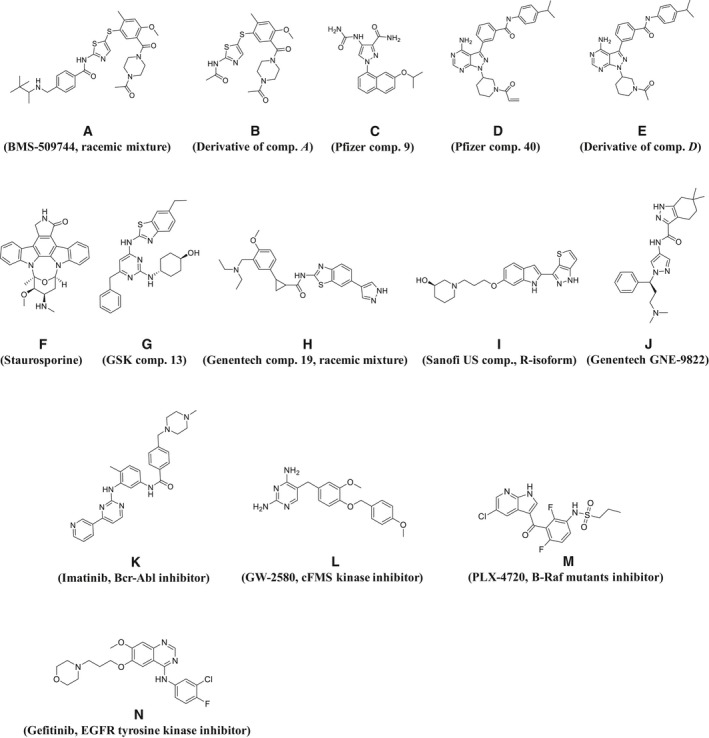
Chemical structures of reference compounds. Compounds *A*–*E* are inactive conformation binders, compounds *F*–*J* are active conformation binders, and compounds *K*–*N* are nonbinders.

Known inactive conformation inhibitors for ITK, BMS‐509744 17, 18 (compound *A*), Pfizer compound 9 19 (compound *C*, allosteric binder) and Pfizer compound 40 20 (compound *D*, covalent binder; Fig. 2), were selected as positive control compounds for SHG assay development. To expand the tool compounds, two more inactive conformational binders were synthesized from two of the known inactive conformational binders above, as follows. Compound *B* was synthesized by deleting the groups of compound *A* that are exposed to solvent, and compound *E* was synthesized by converting the acryloyl group of compound *D* to an acetyl group, rendering this molecule noncovalent. In addition, we selected staurosporine (compound *F*), GSK compound 13 21 (compound *G*), Genentech compound 19 22 (compound *H*), Sanofi US compound 23, 24 (compound *I*), and Genentech GNE‐9822 25 (compound *J*) as active conformation inhibitors, and imatinib 26 (compound *K*), GW‐2580 27 (compound *L*), PLX‐4720 28 (compound *M*) and gefitinib 29 (compound *N*) as nonbinders.

### Cloning, expression, and purification of human tyrosine kinase

The His_6_‐tagged human wild‐type ITK kinase domain (WT ITK) was expressed from the pVL1393/His‐hITK KD vector. This vector was constructed by cloning the human ITK KD from the human ITK full‐length cDNA (OriGene, Rockville, MD, USA) into pVL1393 (Thermo Fisher Scientific, Waltham, MA, USA) using primers that encoded an N‐terminal His_6_‐tag.

His_6_‐tagged human mutant ITK KD (ITK Mutant A) was expressed from the pVL1393/His‐hITK KD MutA vector. This vector was constructed by PCR‐based site‐directed mutagenesis of pVL1393/His‐hITK KD to incorporate the following changes to the native sequence: one native solvent‐exposed amino acid on the αC‐helix was substituted with a cysteine, and the native cysteines (C442, C477) in the WT enzyme were mutated to serines. The myc‐tagged human YopH was cloned utilizing primers that encoded an N‐terminal myc‐tag to amplify human YopH from commercially available cDNA (GenScript, Piscataway, NJ, USA). The resulting PCR product was cloned into pVL1393 resulting in expression vector pVL1393/myc‐YopH. All plasmids were confirmed by sequence analysis.

Recombinant baculovirus was produced using the BaculoGold Transfection Kit (BD Biosciences, San Jose, CA, USA). For protein production, the cells were coinfected with either pVL1393/His‐hITK KD or pVL1393/His‐hITK KD MutA baculovirus, and pVL1393/YopH baculovirus at an appropriate rate to ensure uniform dephosphorylation. Cells were harvested by centrifugation 72 h postinfection and stored at −80 °C.

Wild‐type ITK and ITK Mutant A were purified from cell lysate on Ni‐NTA Superflow (Qiagen, Germantown, MD, USA) and eluted with 50 mm HEPES, pH 8.0, 150 mm NaCl, and 500 mm imidazole. The WT ITK and ITK Mutant A containing fractions were pooled, respectively, and further purified by ion exchange over Q Sepharose Fast Flow (GE Healthcare), followed by gel filtration over a Superdex 200 10/300 GL (GE Healthcare). The final protein buffer contained 50 mm HEPES, pH 8.0, 500 mm NaCl, 10% glycerol, and 1 mm DTT. The protein was concentrated to ~ 2.5 mg·mL^−1^, flash‐frozen with liquid nitrogen, and stored at −80 °C.

Dephosphorylation of WT ITK and ITK Mutant A by YopH was confirmed by western blotting using the monoclonal phosphotyrosine antibody clone 4G10 from Merck Millipore (Darmstadt, Germany).

### ADP‐Glo™ inhibitory assay

Enzymatic activity of WT ITK and ITK Mutant A was measured using the ADP‐Glo™ Kinase Assay 30 (Promega). Activity measurements were taken following the manufacturer's protocols. The ADP production was measured in assay buffer (25 mm HEPES, pH 7.5, 25 mm KCl, 10 mm MgCl_2_, 1 mm DTT, 0.005% Tween 20, 0.1% BSA) in a 384‐well plate format. The reactions were carried out at room temperature in a total volume of 5 μL for 60 min with 70 μm ATP and at excess substrate (SLP76 18) concentrations. ADP‐Glo™ reagent was added at room temperature for 40 min to stop the kinase reaction and deplete the unconsumed ATP, leaving only ADP and a very low background of ATP. Furthermore, kinase detection reagent was added to convert ADP to ATP and introduce luciferase and luciferin to detect ATP. The plate was read by a Paradigm (Molecular Devices, LLC, Sunnyvale, CA, USA) in luminescent mode.

### SPR assay

Wild‐type ITK was immobilized onto an NTA sensor chip using the capture coupling method that results in the capture in a nonrandom orientation by the His_6_‐tag prior to amine coupling to produce functional and stable surfaces 31. The surface of the sensor chip was activated with a mixture of 0.1 mol·L^−1^ *N*‐hydroxysuccinimide and 0.4 mol·L^−1^ *N*‐ethyl‐*N’*‐(dimethylaminopropyl) carbodiimide for 5 min at 10 °C. WT ITK (10 μg·mL^−1^) in running buffer (25 mm HEPES, pH 7.5, 25 mm KCl, 10 mm MgCl_2_, 1 mm DTT, 0.005% Tween 20) was injected for 10 min. Typical immobilization levels ranged from 2900 to 4900 resonance units.

The compounds were diluted directly into the running buffer containing 5% DMSO. Serially diluted compounds were injected in a separate cycle for 60‐s association followed by 60‐s dissociation at a flow rate of 30 μL·min^−1^. Competition experiments were carried out in the presence of ADP at a 1000 μm concentration diluted in the running buffer.

All data analysis was performed using Biacore T200 or Biacore 4000 evaluation software. Sensorgram data were solvent‐corrected and double‐referenced. For kinetic analysis, data were fit to 1 : 1 interaction model. For steady‐state analysis, responses at equilibrium were plotted against the compound concentration and fit to a 1 : 1 Langmuir binding isotherm.

### SHG assay protein labeling

Interleukin‐2‐inducible T‐cell kinase Mutant A was labeled with SHG2‐maleimide (thiol‐reactive dye) in 50 mm sodium phosphate, pH 6.5, 150 mm NaCl, and 10% glycerol. One hundred micromolar of protein was incubated with 500 μm of dye at room temperature for 90 min. The reactions were then centrifuged at 4 °C for 20 min before the reaction was stopped by removing excess dye by buffer exchange with a Zeba™ column (Thermo Fisher) into storage buffer (50 mm HEPES, pH 8.0, 500 mm NaCl, 10% glycerol, 1 mm DTT). The degree of labeling was measured by UV/Vis spectroscopy and was measured to be 1.3 (dyes/protein). The resulting conjugate, ITK Mutant A‐SHG2, was flash‐frozen in liquid nitrogen and stored at −80 °C until use.

### SHG assay for tool compounds using ITK Mutant A‐SHG2

**Figure 3:**
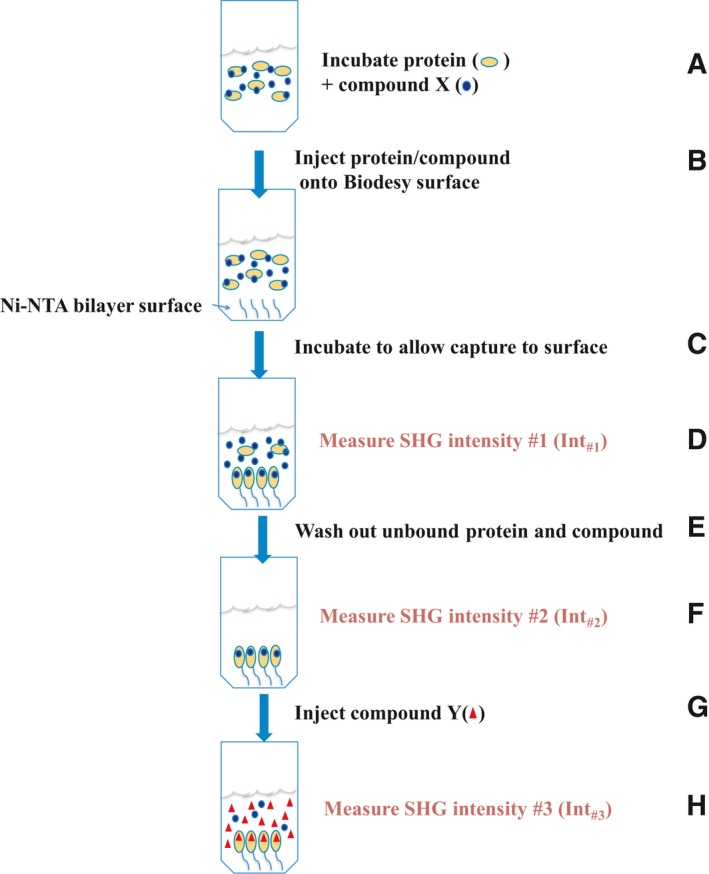
Schematic of the SHG experiment. SHG assay workflow (preincubation and competition methods). (A) Incubate His_6_‐tagged, labeled protein with compound at room temperature to allow binding to come to equilibrium. (B) Inject protein/compound mixture onto the Biodesy Ni/NTA bilayer surface in the wells of a Biodesy Delta 384‐well plate. (C) Allow the capture of the protein/compound to the surface through the Ni/NTA:His_6_‐tag interaction by incubation at room temperature. (D) Measure SHG signal intensity. (E) Wash out unbound protein/compound. (F) Measure SHG signal intensity. (G) Inject a second compound. (H) Measure the change in SHG signal intensity to determine whether the protein has changed conformation.

Five microliter of ITK Mutant A labeled with SHG2‐maleimide (ITK Mutant A‐SHG2) was preincubated at room temperature with 20 μm compound for 60 min in assay buffer (25 mm HEPES, pH 7.5, 25 mm KCl, 10 mm MgCl_2_, 1 mm DTT, 0.005% Tween 20). 10 μL of the protein/compound mixture was added to 10 μL of assay buffer on the Ni/NTA bilayer surface. Protein was allowed to tether to the surface through the Ni/NTA:His_6_‐tag interaction at room temperature for 90 min, at which point the SHG signal was measured (Fig. 3A–D).

In a competition assay, after SHG readout #1 (Fig. 3D), unbound protein and excess compound were washed out, and then, SHG baseline signal was measured (Fig. 3F). The washed well was then injected with 20 μL of 10 μm test compound, and the SHG signal was measured again (Fig. 3H). The time between measurement of SHG intensity #2 (Fig. 3F) and measurement of SHG intensity #3 (Fig. 3H) was 5 min.

To calculate the percent change in SHG intensity (% Δ_SHG_), the SHG intensity of readout #2 (Int_#2_) was subtracted from the SHG intensity of readout #3 (Int_#3_) and then divided by the initial second‐harmonic intensity (Int_#2_) according to the following equation:

$$\%\Delta_{\mathrm{SHG}}=\left({\text{Int}}_{\#3}-{\text{Int}}_{\#2}\right)/{\text{Int}}_{\#2}.$$

### SHG assay for binning HTS hits

The compounds to be analyzed were mixed with 500 nm ITK Mutant A‐SHG2 and allowed to incubate at room temperature for 60 min. The protein/compound mixtures were added to the Ni/NTA bilayer surface with a 1 : 1 dilution for a final protein concentration of 250 nm in a 384‐well plate format. Each compound was run at 2.5, 10, and 40 μm for lower potency compounds (IC_50_ values ≥ 1 μm) or at 0.31, 1.3, and 5.0 μm for higher potency compounds (IC_50_ values of < 1 μm). Protein/compound mixtures were allowed to attach to the bilayer surface for 90 min at room temperature, and the SHG signal was then measured (Fig. 3D). All experiments were performed in triplicate. SHG signal is reported in photon counts per second (cps).

## Results

### Characterization of protein and tool compounds

**Figure 4:**
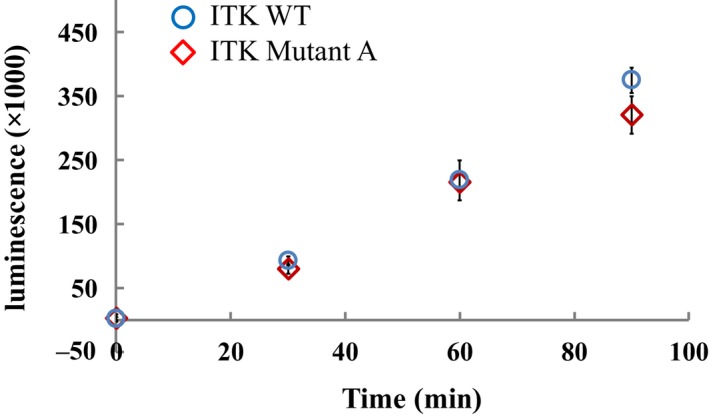
Enzyme activity of WT ITK and ITK Mutant A. The time course of the phosphorylation reaction of WT ITK (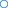) and ITK Mutant A (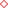) is similar, indicating that the mutations made to the WT sequence do not alter the activity of the resulting mutant protein. Data were recorded for 90 min using ADP‐Glo™ Kinase Assay and are shown as the mean ± SD of triplicate measurements.

**Figure 5:**
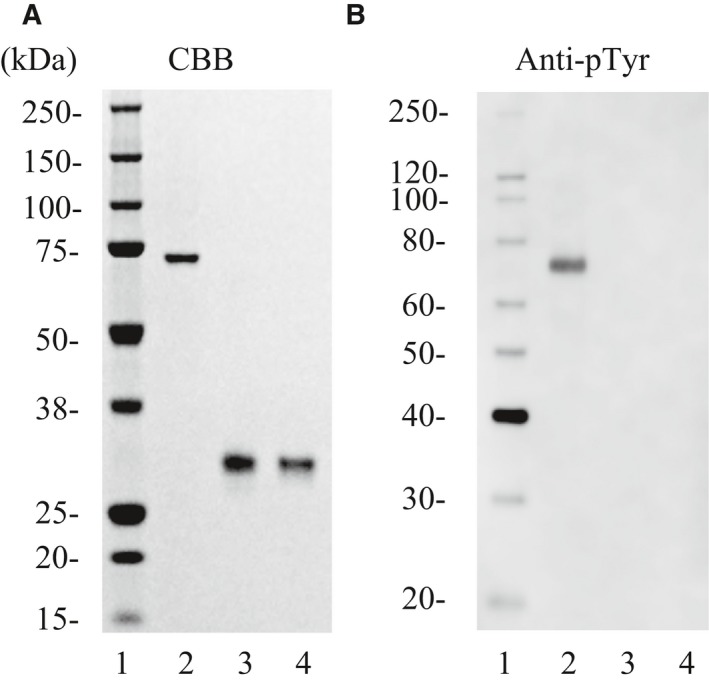
SDS/PAGE and western blotting of WT ITK and ITK Mutant A. (A) Purified kinases were analyzed on SDS/PAGE, stained with Coomassie blue. (B) Western blotting results with antiphosphotyrosine. Lanes 1, molecular weight markers; lanes 2, YopH phosphatase untreated full‐length ITK (this was applied as a reference of phosphorylated kinase); lanes 3, dephosphorylated WT ITK; lanes 4, dephosphorylated ITK Mutant A.

His_6_‐tagged human ITK KD (WT ITK) was utilized for both the biochemical and surface plasmon resonance (SPR) assays. WT ITK with a cysteine substituted in the αC‐helix (ITK Mutant A) was used for the SHG assay. The engineered Cys residue was inserted in order to conjugate the dye to a site‐specific location that reports on the structural change between the active and inactive conformations of the protein. The amino acid substitutions in ITK Mutant A (C442S/C477S, and Cys substitution on αC‐helix) were shown to have no impact on enzymatic activity (Fig. 4). To allow the protein to adopt both an active and an inactive conformation, all proteins were coexpressed with the tyrosine phosphatase, YopH, in baculovirus to fully dephosphorylate them. Dephosphorylation was validated by western blot using a monoclonal phosphotyrosine antibody (Fig. 5). We prepared 14 tool compounds with known biochemical ITK inhibitory activity and binding affinity summarized in Fig. 2 and Table 1, respectively.

**Table 1:** Biochemical inhibitory activity and binding affinity of tool compounds

No	Form	Company	Inhibitor	IC_50_ (m)	*K*_D_ (m)
*A*	Inactive	BMS	BMS‐509744	2.5 × 10^−9^	1.3 × 10^−9^
*B*	Inactive		Derivative of A	2.6 × 10^−7^	7.5 × 10^−8^
*C*	Inactive	Pfizer	Comp 9	2.4 × 10^−8^	2.5 × 10^−8^
*D*	Inactive	Pfizer	Comp 40	8.5 × 10^−9^	NT
*E*	Inactive		Derivative of D	4.6 × 10^−7^	1.1 × 10^−6^
*F*	Active		Staurosporine	1.3 × 10^−8^	1.2 × 10^−8^
*G*	Active	GSK	Comp 13	3.5 × 10^−9^	3.6 × 10^−9^
*H*	Active	Genentech	Comp 19	1.3 × 10^−9^	2.1 × 10^−9^
*I*	Active	Sanofi US		1.8 × 10^−9^	1.5 × 10^−9^
*J*	Active	Genentech	GNE‐9822	1.1 × 10^−9^	3.5 × 10^−9^
*K*	Nonbinder	Novartis	Imatinib	> 3.0 × 10^−5^	> 3.0 × 10^−5^
*L*	Nonbinder	GSK	GW‐2580	> 3.0 × 10^−5^	> 3.0 × 10^−5^
*M*	Nonbinder	Plexxikon	PLX‐4720	> 3.0 × 10^−5^	> 3.0 × 10^−5^
*N*	Nonbinder	AstraZeneca	Gefitinib	> 3.0 × 10^−5^	> 3.0 × 10^−5^

Inhibitory activities and affinities of each compound against His_6_‐tagged human ITK KD (WT ITK) were measured by ADP‐Glo™ Kinase Assay and SPR assay, respectively.

*K*_D_ of compound *D* was not measured due to covalent inhibitor.

### SHG assay development

**Figure 6:**
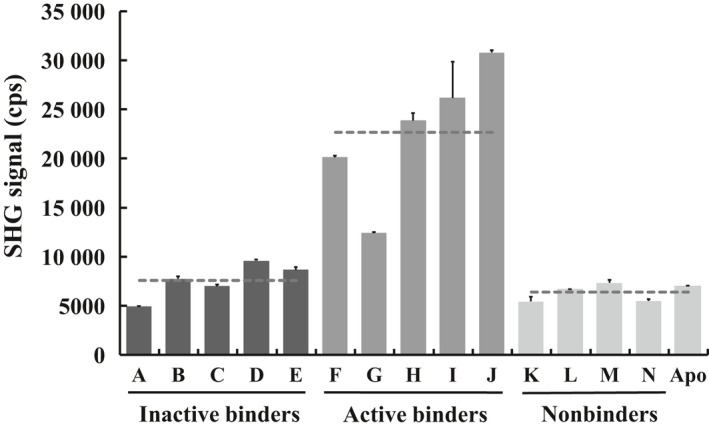
SHG signal of ITK Mutant A‐SHG2 with tool compounds. Compounds *A*–*E* are inactive conformation inhibitors and show low SHG intensity compared to compounds *F*–*J* which are active conformation inhibitors. Compounds *K*–*N* are nonbinders and cannot be distinguished from inactive conformation inhibitors, but are clearly different than active conformation inhibitors. Compound *G* shows an intermediate SHG intensity most likely due to low solubility of this compound. Dashed lines are the respective averages of inactive conformation inhibitors, active conformation inhibitors, and nonbinders. Data are shown as the mean ± SD of duplicate measurements.

Interleukin‐2‐inducible T‐cell kinase Mutant A‐SHG2 was preincubated with each of the 14 tool compounds to demonstrate that we could distinguish inactive conformation inhibitors, active conformation inhibitors, and nonbinders by SHG signal differences. A schematic of the assay is shown in Fig. 3A–D. The results of this panel showed large SHG signal compared to apo protein for compounds *F*,* H*,* I*, and *J* (Fig. 6), all of which are known active conformation inhibitors. Compound *G* is also an active conformation inhibitor but showed an intermediate SHG signal, which could be due to its lower solubility. All other compounds in the panel showed similar SHG signal intensity to the apo form of the protein. Compounds *A*–*E* are known inactive conformation inhibitors, and compounds *K*–*N* are known nonbinders. These two classes of compounds cannot be distinguished from one another in the current assay setup. However, they can be readily distinguished from the active conformation inhibitors.

### SHG assay validation

To show that ITK Mutant A‐SHG2 maintained its activity on the surface, compound injection after immobilization of the protein was performed. A schematic of the assay is shown in Fig. 3A–H. ITK Mutant A‐SHG2 was preincubated with compound *E*, a low‐affinity inactive conformation inhibitor (*K*_D_ of 1.1 μm for WT ITK). The protein/compound mixture was injected onto the Ni/NTA bilayer surface and allowed to tether to the surface. Unbound protein/compound was washed out, and then, a high‐affinity inactive conformation inhibitor (compound *A, K*_D_ of 1.3 nm for WT ITK), a high‐affinity active conformation inhibitor (compound *J, K*_D_ of 3.5 nm for WT ITK) or buffer, was injected onto the complex on the surface.

**Figure 7:**
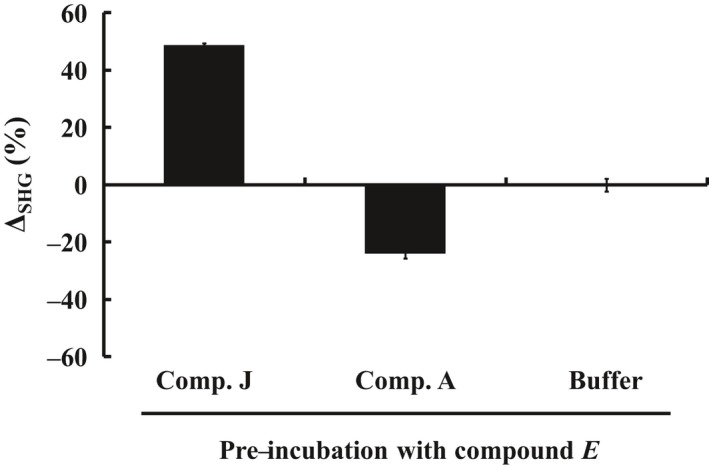
ITK Mutant A is free to change conformation when tethered to the Ni/NTA bilayer surface. ITK Mutant A was preincubated with control compound *E* and then tethered to the Ni/NTA bilayer surface. Compound *A* (inactive conformation inhibitor) and compound *J* (active conformation inhibitor) were injected onto compound *E* bound ITK Mutant A on the surface. Observation of a negative SHG intensity change after compound *A* injection, and a positive SHG intensity change after compound *J* injection, suggests movement of the protein into the inactive and active conformations, respectively. Data are shown as the mean ± SD of duplicate measurements.

Upon compound *A* injection, the SHG signal decreased by 24%, suggesting that more conjugate molecules shifted to the inactive conformation (Fig. 7). When compound *J* was injected, there was a dramatic increase of 49% in SHG signal, suggesting a switch from the inactive to the active conformation. The observed SHG signal change correlates with the preincubation results in Fig. 6, which shows relatively low SHG signal for inactive conformation inhibitors and high SHG signal for active conformation inhibitors. The signal changes upon competitive binding with the high‐affinity inhibitor also demonstrate that ITK Mutant A‐SHG2, when stabilized by preincubation with compound *E*, remains functional on the surface. Injection of buffer showed a decrease in the SHG intensity of −1.7% indicating no conformational change.

### Optimization of protein concentration for a more high‐throughput SHG assay

**Figure 8:**
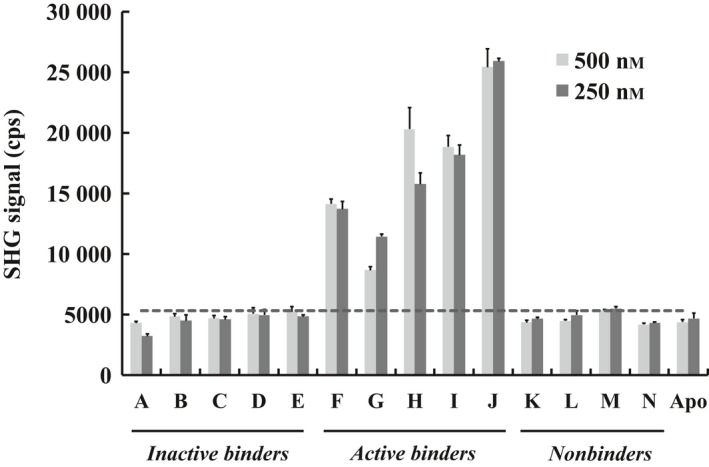
SHG signal of ITK Mutant A‐SHG2 with compound at two protein concentrations. SHG signals were measured at 250 and 500 nm of protein with 10 μm of compound to find the optimal protein concentration for the binning experiment. The dashed line (5300 cps) corresponds to +3σ of 250 nm protein with 10 μm compound *E*. Data are shown as the mean ± SD of triplicate measurements.

To develop a high‐throughput SHG screen, we attempted to reduce the labeled protein concentration in the assay to conserve material. During the initial phase of assay development, we incubated 5 μm ITK Mutant A‐SHG2 with 20 μm compound. This reaction was then diluted 1 : 1 with assay buffer, and the final concentrations of protein and compound were 2.5 and 10 μm, respectively. Holding the compound concentration constant, we tested final protein concentrations of 500 and 250 nm. Both protein concentrations gave similar results with SHG signals of active conformation inhibitors above three times the standard deviation (+3σ) of the signal level (5300 cps) of the protein bound to compound *E* (Fig. 8). Therefore, we performed the binning experiments at 250 nm final protein concentration (160 ng per well), and a +3σ difference of the signal bound to compound *E,* the lowest affinity inactive form binder among the tool compounds, was used as a criterion for separation between active conformation inhibitors and inactive conformation inhibitors/nonbinders.

### Conformational binning of hits from a HTS with the SHG assay

Next, we used the optimized SHG assay to distinguish between active and inactive conformation inhibitors within the set of 105 hits previously identified in a high‐throughput biochemical screen; the screen only identified whether they are inhibitors but did not provide any information about their conformational mechanism of action (MOA). Inhibitory activity of these compounds was lower than 10 μm, and most of these binders showed a typical 1 : 1 interaction and stoichiometry by SPR.

**Figure 9:**
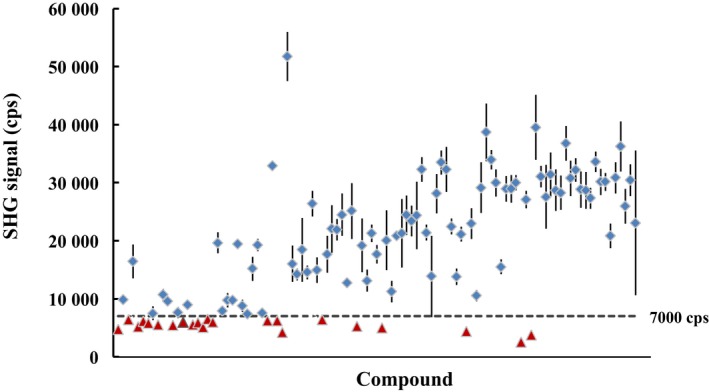
SHG signal intensity for conformational binning of HTS hits. A total of 105 compounds from the HTS biochemical screen were analyzed with the SHG assay in triplicate. Dashed line (7000 cps) was +3σ of the SHG signal of ITK Mutant A‐SHG2 bound to compound *E* and was used as the cutoff to distinguish between active (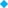) and inactive (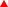) conformation inhibitors. Of the 105 compounds analyzed, 22 bin as inactive conformation inhibitors. Data are shown as the mean ± SD of triplicate measurements at the highest inhibitor concentration (5 or 40 μm).

**Figure 10:**
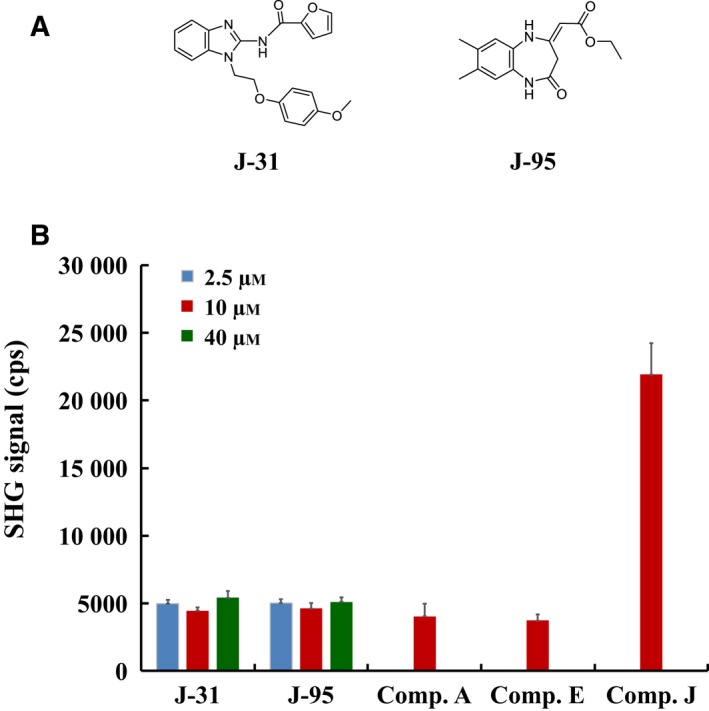
Conformational binning of J‐31 and J‐95 by SHG assay. (A) Chemical structures of J‐31 and J‐95. (B) SHG signals were measured at 2.5, 10, and 40 μm of J‐31 or J‐95 and at 10 μm of compounds *A*,* E*, or *J*. Control compounds *A* and *E* show low SHG signal intensity compared to control compound *J*. Compounds *A* and *E* are known inactive conformation inhibitors and compound *J* is a known active conformation inhibitor, both bins are clearly distinguished by SHG signal intensity. Test compounds J‐31 and J‐95 show SHG signal intensities similar to control compounds *A* and *E* and can therefore be binned as inactive conformation inhibitors. Data are shown as the mean ± SD of triplicate measurements.

The compounds were classified into low potency (IC_50_ values ≥ 1 μm) and high potency (IC_50_ values < 1 μm) molecules. Each compound was evaluated at 2.5, 10, and 40 μm for low potency or at 0.31, 1.3, and 5.0 μm for high potency compounds. The results of the screen showed a clear difference between active and inactive conformation inhibitors (Fig. 9). Compounds were classified as active conformation inhibitors when the resulting SHG signal was above the +3σ signal (7000 cps) of the control compound (compound *E*) at the highest inhibitor concentration. In contrast, compounds below the +3σ of compound *E* were classified as inactive conformation inhibitors, low‐affinity active conformation inhibitors, or nonbinders. Of 105 compounds, 22 were determined to be inactive conformation inhibitors, weak active conformation inhibitors, or nonbinders. However, based on the HTS assay results and SPR follow‐up (data not shown), all 105 compounds were shown to bind ITK, with the affinity of the weakest binders at ~ 10 μm. Therefore, all 22 compounds were neither weak active conformation inhibitors nor nonbinders, suggesting that these compounds with low SHG signal were inactive conformation inhibitors. As examples, SHG signals of J‐31 (IC_50_ of 5.5 μm for WT ITK) and J‐95 (IC_50_ of 3.3 μm for WT ITK) are shown in Fig. 10. SHG signals at all concentrations of these compounds are comparable in intensity to compounds *A* and *E*, indicating that they too are inactive conformation inhibitors.

### Kinase selectivity of ITK inhibitors

Having determined by SHG that J‐31 and J‐95 are inactive conformation inhibitors, we next evaluated their selectivity in a broad kinase panel using biochemical assays. We screened against 54 kinases in the DiscoveRx KINOME*scan*® (Eurofins DiscoverX, San Diego, CA, USA) at a concentration of 50 μm of J‐31 and 20 μm of J‐95. J‐95 was assessed at 20 μm due to low solubility. J‐31 only inhibited one kinase (CLK1) at > 65% and J‐95 only inhibited two kinases (HCK and LCK) at > 65% (Table 2), indicating good specificity of these compounds.

**Table 2:** Kinase selectivity of J‐31 and J‐95 as assessed by DiscoveRx KINOME*scan* ^®^

DiscoveRx gene symbol	Percentage of control binding	
	J‐31	J‐95
AKT1	100	85
ASK1	100	100
AURKB	80	96
BMPR1B	72	74
BRAF	88	100
BRK	100	100
CAMK1	84	86
CAMK2A	97	100
CDK2	90	100
CDKL2	100	100
CLK1	21	100
CSNK1D	79	98
DAPK3	98	96
DRAK2	100	100
DYRK2	68	100
EGFR	100	47
EPHA3	86	92
EPHB4	83	100
FES	89	100
FGFR1	95	80
GRK4	91	84
HCK	86	33
IKK‐beta	63	100
IRAK4	79	87
JAK2(JH1 domain‐catalytic)	53	61
KIT	41	100
LCK	98	29
MAP4K5	95	99
MARK2	82	82
MAST1	47	90
MEK6	97	95
MERTK	90	100
MRCKA	98	100
NEK3	63	79
p38‐alpha	87	97
PAK4	100	94
PCTK1	84	98
PKAC‐alpha	93	97
PKMYT1	93	96
PLK2	69	93
PRKCH	100	100
PRKD1	100	100
RSK1(Kin.Dom.1‐N‐terminal)	92	99
RSK4(Kin.Dom.2‐C‐terminal)	76	95
SGK3	72	90
SLK	100	100
SYK	100	61
TAOK1	100	91
TEC	100	86
TIE2	100	100
TRKC	100	97
TSSK1B	100	100
ULK1	75	97
YANK1	92	94

J‐31 and J‐95 were screened at 50 and 20 μm, and the results for the primary screen binding interactions are reported as a percentage of control binding, where lower numbers indicate stronger hits in the matrix. Values of < 35% control binding, indicating > 65% inhibition are considered ?hits’. The hit of J‐31 was CLK1 at 21% control binding, indicating 79% inhibition. The hits of J‐95 were HCK and LCK at 33 and 29% control binding, indicating 67 and 71% inhibition, respectively.

### Determination of binding site

**Figure 11:**
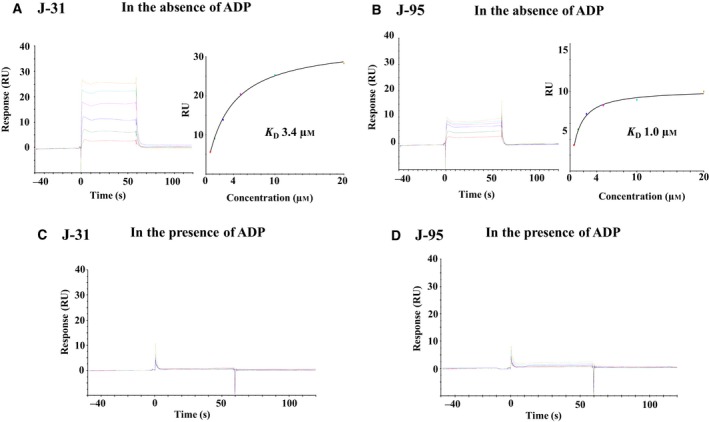
SPR data of J‐31 and J‐95. (A,B) Left: J‐31 and J‐95 were injected on the surface of WT ITK at a range of concentrations (20 μm and twofold dilutions thereof). Right: Nonlinear curve fitting using a 1 : 1 Langmuir binding isotherm yielded *K*_D_ of 3.4 μm and 1.0 μm. (C,D) J‐31 and J‐95 were injected on the surface of WT ITK at a range of concentrations (20 μm and twofold dilutions thereof) in the presence of 1000 μm ADP. J‐31 and J‐95 did not bind in the presence of ADP suggesting that both compounds are ATP‐site binders.

To help identify potential binding sites on ITK and provide valuable information for further optimization, we used the Biacore assay to examine competition between the inhibitors and ADP. J‐31 and J‐95 bound to ITK in the absence of ADP (*K*_D_ of 3.4 and 1.0 μm, respectively) and did not bind in the presence of ADP (Fig. 11). Therefore, both compounds would probably be ATP‐site binders that stabilize the inactive conformation of ITK.

## Discussion

Second‐harmonic generation is a nonlinear optical technique in which two photons of equal energy are combined by a nonlinear material or molecule to generate one photon with twice the energy 32, 33. Recently, the SHG technique has been shown to be a powerful method for studying biomolecular structure and conformational changes. In this study, we developed an SHG‐based assay to identify compounds that stabilized the ITK αC‐helix Glu‐out inactive conformation without the need to determine costructures by X‐ray crystallography. From among a set of 105 HTS hits, we identified 22 inactive conformation inhibitors by excluding compounds with SHG signal comparable to the active conformation inhibitor control.

It is important for precise compound binning that the SHG‐active dye is conjugated at a suitable site on the target protein and that the activity of an immobilized target protein is maintained. Multiple approaches can be used to label proteins. Generally WT proteins can be labeled randomly at lysines through amine‐reactive chemistry. In parallel, mutant proteins with engineered cysteines can be labeled through thiol‐reactive chemistry to introduce dyes to targeted residues. Although most published assays of SHG‐detected conformational changes rely on random labeling 9, we adopted a site‐specific labeling strategy to distinguish inactive conformation inhibitors. To select the site of the cysteine mutant for introducing the SHG dye, we focused on movements of the αC‐helix. This helix exhibits a large movement between the inactive and active conformations in the reported ITK X‐ray structures (Protein Data Bank ID code: 4L7S 21, 4MF1 22, 4PQN 25 for active conformations and 3MJ2 17, 4M14 19, 4HCU 20 for inactive conformations). A single cysteine mutant was introduced into the kinase αC‐helix resulting in an SHG assay that reports on the conformational state of the labeled αC‐helix upon binding active or inactive conformation inhibitors. The ITK Mutant A‐SHG2 conjugate was found to discriminate well between the inactive and active conformation inhibitors with a set of 14 tool compounds, proving that site‐specific labeling of an engineered cysteine in the αC‐helix is effective to distinguish active and inactive conformations in this study.

To ascertain whether ITK Mutant A‐SHG2 maintained its activity on the surface during the assay, we ran an order of addition experiment. The SHG assay resolved the displacement of a low‐affinity inactive conformation inhibitor (compound *E, K*_D_ of 1.1 μm for WT ITK) with a high‐affinity inactive conformation inhibitor (compound *A, K*_D_ of 1.3 nm for WT ITK) and a high‐affinity active conformation inhibitor (compound *J, K*_D_ of 3.5 nm for WT ITK; Fig. 7). Therefore, ITK can be stably tethered to the surface and produce functional responses, as expected.

Next, we used the SHG assay to select inactive conformation inhibitors from a set of 105 HTS hits. All compounds were previously validated to bind ITK by Biacore, but could not be binned into conformational class without requiring X‐ray cocrystal structure determination. NMR and X‐ray crystallography have been generally used to detect conformational shifts, but these methods have low to mid‐throughput, require large amounts of sample, and are often challenging. In contrast, the SHG assay is very efficient in terms of protein consumption and throughput. For instance, 500 μg of protein would be required for screening about 100 compounds at three concentrations in a triplicate. Moreover, there is no target protein size limitation with SHG. SHG can be a powerful tool, providing information about ligand‐induced conformational changes.

In the assay format described here we did not attempt to quantitatively measure the affinity of compounds as we used an SPR assay instead. Rather, we focused on selecting inactive form binders from hits qualified and validated by SPR and inhibitory assays. Of 105 compounds, 22 compounds were determined by SHG to be inactive conformation inhibitors, providing crucial structural information about the compound MOA and supplementing the typical screening data consisting of potency and affinity routinely measured by orthogonal methods. J‐31 and J‐95 were selected as examples and were evaluated in a broad kinase panel. Both compounds demonstrated high selectivity for ITK.

The identification of a subset of compounds that stabilize the inactive conformation of ITK among a larger pool of hits using an SHG assay creates an opportunity for rapidly developing novel highly selective kinase inhibitors against this kinase and, in principle, any kinase target in a high‐throughput manner. We believe that applying similar SHG‐based assays opens the way to discover high‐selectivity inhibitors across many other members of the kinome and with other protein classes as well.

## Author contributions

YH, KI, SK, and JS designed and coordinated the experiments. KU and TS prepared the constructs and purified the proteins. RH and YH performed SPR experiments. RH performed kinase inhibitory experiments. TY and KC performed SHG experiments. YH, RH, and TY wrote the manuscript.
